# Creation of an Industrial *Bacillus thuringiensis* Strain With High Melanin Production and UV Tolerance by Gene Editing

**DOI:** 10.3389/fmicb.2022.913715

**Published:** 2022-07-22

**Authors:** Lingyi Zhu, Yawen Chu, Bowen Zhang, Ximu Yuan, Kai Wang, Zhiyu Liu, Ming Sun

**Affiliations:** ^1^State Key Laboratory of Agricultural Microbiology, Hubei Hongshan Laboratory, College of Life Science and Technology, Huazhong Agricultural University, Wuhan, China; ^2^Hubei Shuiguohu Senior High School, Wuhan, China

**Keywords:** *Bacillus thuringiensis*, melanin, ultraviolet radiation, insecticidal activity, biopesticide

## Abstract

*Bacillus thuringiensis* produces insecticidal crystal proteins (ICPs) which exhibit strong insecticidal toxicity. But when used in the field, ICPs would be destroyed by ultraviolet (UV) radiation in sunlight, thus decreasing the insecticidal activity and shortening the persistence. To improve the duration of *B. thuringiensis* preparations, we endowed a highly toxic industrial *B. thuringiensis* HD-1 with UV tolerance by making it produce melanin, a pigment that absorbs UV radiation. In *B. thuringiensis*, melanin is derived from homogentisate (HGA), an intermediate in the tyrosine pathway. And the absence of homogentisate-1,2-dioxygenase (HmgA) will lead to the formation of melanin. In this study, we used the CRISPR/Cas9 system to knock out the *hmgA* gene and obtained a melanin-producing mutant HD-1-Δ*hmgA* from strain HD-1. The melanin yield by mutant HD-1-Δ*hmgA* reached 3.60 mg/mL. And the anti-UV test showed that melanin serves as a protection to both the organism and the ICPs. After UV irradiation for 3 h, mutant HD-1-Δ*hmgA* still had an 80% insecticidal activity against the cotton bollworm, *Helicoverpa armigera*, while the control line only had about 20%. This study creates a light-stable biopesticide prototype based on a classic industrial strain that can be applied directly and takes the melanin-producing strain as a concept to improve the preparation validity.

## Introduction

*Bacillus thuringiensis* is a Gram-positive, spore-performing, soil bacterium widely found in nature. Its main feature is the production of typically shaped crystals during sporulation. The insecticidal crystal proteins (ICPs), also known as δ-endotoxins, are mostly coded by *cry* (crystal) or *cyt* (cytolytic) genes (Crickmore et al., 1998). ICPs are selectively poisonous to various insect orders including Lepidoptera, Coleoptera, Diptera, and other species like nematodes, mites, and protozoa (Schnepf et al., 1998). Therefore, *B. thuringiensis* is an ideal biological control agent and is extensively used in the fields of agriculture, forestry, and mosquito control (Schnepf et al., 1998). Compared with chemical pesticides, *B. thuringiensis* preparations have specific toxic effects on target organisms, pose no threat to human health, and are environmentally friendly. Also, various transgenic crops expressing insecticidal toxins have been grown worldwide (Kumar et al., 2008). The research on *B. thuringiensis* has promoted the development of ecological agriculture greatly.

Although *B. thuringiensis* preparations have become the most successful biopesticide in the world, several shortcomings still hinder their application. They are susceptible to a series of environmental factors, such as sunlight, rainfall, dew, soil pH, and temperature under field condition (Brar et al., 2006), among which UV rays in sunlight is the most important factor. UV-B (280–310 nm) and UV-A (320–400 nm) will cause degradation of ICPs and reduce the insecticidal ability (Sanchis et al., 1999). Usually, after 1 day of exposure to sunlight, *B. thuringiensis* products will be rapidly inactivated, but it usually takes 2–3 days to bring the insecticidal effects into full play. Hence the cost of these products rises as repeated spraying is necessary (Sansinenea and Ortiz, 2015). Therefore, it is imminent to create light-stable *B. thuringiensis* preparations with higher insecticidal efficiency.

Researchers have proposed a series of approaches to solving this problem. Some expressed the *cry* gene in *Pseudomonas fluorescens* and *Anabaena* to lower the damage from UV light (Khasdan et al., 2003; Peng et al., 2003). Studies showed that the olive mill wastewater can protect *B. thuringiensis* spores. Using latex particles, GO nanosheets, olive oil, ethanol, and water to encapsulate *B. thuringiensis* in colloidosomes will improve stability of ICPs under UV-A radiation (Jallouli et al., 2014; Jalali et al., 2020). Also, the external addition of UV protective agents such as methyl green and rhodamine B can absorb UV rays, thereby protecting spores from the light (Cohen et al., 1991). However, due to the cost and practicability, these methods have not been popularized.

Melanins, a natural sunscreen that absorbs the broadband of UV–visible light spectrum (Tran-Ly et al., 2020), is an ideal photoprotective pigment that attracts researchers all the time. They are biopolymers derived by the oxidation of phenols and subsequent polymerization of intermediate phenols and their resulting quinones (Solano, 2014). Melanins are widely found in prokaryotes and eukaryotes (Choi, 2021), and can be classified into three main categories based on their structural monomers: eumalanins, pheomelanins, and allomelanins (Plonka and Grabacka, 2006). Both eumalanins and pheomelanins are derived from the oxidation of tyrosine or phenylalanine to o-dihydroxyphenylalanine (DOPA) and dopaquinone. Allomelanins, however, are derived from the oxidation of nitrogen-free diphenols, such as catechol, 1,8-dihydroxynaphtalene, γ-glutaminyl-3,4-dihydroxybenzene, homogentisic acid (HGA) as well as 4-hydroxyphenyl acetic acid (Plonka and Grabacka, 2006; Singh et al., 2021). HGA derived from tyrosine or phenylalanine are further catalyzed by homogentisate-1,2-dioxygenase (HmgA) to acetoacetic acid and fumaric acid (Turick et al., 2010; Ahmad et al., 2017). But when HmgA is inactivated or absent, HGA will accumulate and secrete out of the cell, then self-oxidize and polymerize to form pyomelanin (Rodríguez-Rojas et al., 2009).

It is demonstrated that the addition of melanin can also protect *B. thuringiensis* from UV lights (Liu et al., 1993; Ruan et al., 2004; Sansinenea and Ortiz, 2015). But it would be more cost-saving if *B. thuringiensis* can produce melanin spontaneously. It is reported that *B. thuringiensis* subsp. *Dendrolimus* L-7601 produces melanin naturally (Chen et al., 2004), many researches were further conducted to reveal the mechanism. In a screening process of *B. thuringiensis* mutagenesis, Ruan et al. found that most *B. thuringiensis* strains have the potential to produce melanin in the presence of L-tyrosine at high temperatures (42°C), but the insecticidal proteins could not be synthesized under this condition (Ruan et al., 2004). Sub-culturing at 42°C, Liu et al. obtained a mutant strain BMB181 with high melanin production from crystalliferous strain BMB171 (Liu et al., 2013). Later studies showed that the pigment produced by strain BMB181 was derived from the HGA pathway, and the elevated temperature caused a single amino acid substitution in HmgA, leading to its deactivation, which was responsible for melanin overproduction in *B. thuringiensis* (Yang et al., 2018). The same pathway also worked in *B. thuringiensis* L-7601 (Cao et al., 2018). Subsequently, Tan et al. constructed an *hmgA*-deletion mutant from strain BMB171, which gained the ability to produce pyomelanin (Tan et al., 2019). Although these strains can produce melanin, they are crystalliferous with no insecticidal property, thus having limited application value.

The insecticidal ability and spectra of *Bacillus thuringiensis* vary greatly among different strains, and only the highly toxic strains are used for biopesticide production. *B. thuringiensis* serovar *kurstaki* HD-1, an industrially patented strain with high toxicity, has been used as an effective biopesticide ever since its isolation in 1970 (Zhu et al., 2015). Strain HD-1 is originally used in the microbial insecticide Dipel and has become one of the most famous and successful commercial biopesticides worldwide. But there is little known about melanin production in highly toxic strains.

In this study, we chose *B. thuringiensis* HD-1 as the target then used CRISPR/Cas9 system to knock out the *hmgA* gene and finally obtained a melanin-producing mutant HD-1-Δ*hmgA*. The mutant has a relatively high melanin yield and also shows better resistance to UV light and stronger insecticidal ability than the wild type. The mutant HD-1-Δ*hmgA* has the potential for direct industrial production and serves as a light-stable biopesticide for long-term application. Using this approach, we adopt melanin-producing strains as a concept for improving the duration of *B. thuringiensis* preparations. Also, this work is a continuation of the previous research by Yang et al., extending the research at a technical level.

## Method

### Bacterial Strains, Plasmids, and Growth Conditions

The bacterial strains and plasmids used in this study are shown in Table 1. Bacteria were grown in Luria-Bertani (LB) medium at 37°C (*E. coli*) or 28°C (*B. thuringiensis*) with shaking at 200 rpm. Appropriate antibiotics were added at the following concentrations: 25 μg/mL erythromycin (Erm), 50 μg/mL kanamycin (Kan). ICPM medium (1L) is needed for crystal-forming: peptone 6 g, glucose 5 g, CaCO_3_ 1 g, MgSO_4_ 0.5 g, KH_2_PO_4_ 0.5 g. Artificial diet (1 L) for cotton bollworm: 40 g soybean flour, 20 g yeast extract, 14 mL 30% acetic acid, 5 g vitamin C, 1.5 g sodium benzoate, and 16 g agar powder.

**Table 1:** Strains and plasmids used in this work.

**Strains or plasmids**	**Characteristics**	**Source or reference**
***Escherichia coli***		
DH5α	*supE*44 Δ*lacU*169 (ϕ80*lacZ*ΔM15) *hsdR*17 *recA*1 *endA*1 *gyrA*96 *thi*-1 *relA*1	Stored in this lab
***Bacillus thuringiensis***		
HD-1	Highly toxic strain, serovar *kurstaki*	Zhu et al., 2015
HD-1-*ΔhmgA*	HD-1 derivative with deletion of *hmgA* gene, renamed as strain YBT1173	This work
**Plasmids**		
pJOE8999	CRISPR/Cas9 vector; Kan^r^	Altenbuchner, 2016
sgRNA-PJOE8999	pJOE8999 containing sgRNA sequence; Kan^r^	This work
up-do-sgRNA-PJOE8999	sgRNA-pJOE8999 containing homologous template sequence; Kan^r^	This work

### Construction of *HmgA* Knockout Mutants in Strain HD-1

The plasmid pJOE8999 is a shuttle vector widely used for gene knockout in bacteria. It has a pUC minimal origin of replication for *E. coli*, a temperature-sensitive replication origin of pE194ts for *B. subtilis*, and a kanamycin resistance gene working in both organisms (Altenbuchner, 2016).

The knockout plasmid was constructed as follows: The sgRNA-F/R were designed on CRISPy-web and were added *Bsa* I sites on the 5' end, then self-annealed to form dsRNA. The plasmid pJOE8999 was digested by *Bsa* I, then ligated with sgRNA. Primers HD-up-F/R and HD-do-F/R were used to amplify the upstream and downstream homologous template of *hmgA* from the strain HD-1 genome and were added *Sfi* I sites on the 5' end. The plasmid sgRNA-pJOE8999 and the homologous fragments were digested by *Sfi* I, then ligated together to form up-do-sgRNA-PJOE8999. All oligonucleotide primers used in this study are listed in Table 2.

**Table 2:** Primers used in this work.

**Primers**	**oligonucleotides (5' → 3')**	**Use**
HD-sg-F	TACGGATTCCCCATG GACCGCATC	HD-1-sgRNA in CRISPR vector
HD-sg-R	AAACGATGCGGTCCA TGGGGAATC	
HD-up-F	AAGGCCAACGAGGCC GGGCGAAATATTTCTCGTGA	Amplification of homology template
HD-up-R	AAGGCCATGTTGGCCGCC CATCACCCGCTTCCTTT	
HD-do-F	AAGGCCAACATGGCCGTA AAAAAGGCATGCTCTCA	
HD-do-R	AAGGCCTTATTGGCCTG CCCGAGACAGGAACTGAA	
HD-Y-F	TGGACGAAGA GGATTAGATG	*hmgA* Knockout verification
HD-Y-R	CGAGACAGGA ACTGAAGAA	

*The underlined sequences are the restriction sites*.

The plasmid up-do-sgRNA-PJOE8999 was then transformed into *B. thuringiensis* HD-1 by electroporation. The transformants were picked onto a new LB plate containing 50 μg/mL kanamycin, then continued to subculture. If the colonies became brownish after 2–3 days, they were considered possible candidates for *hmgA* deletion mutants. Primers HD-Y-F and HD-Y-R were used to verify the knockout mutant, and the correct ones were confirmed by DNA sequencing.

### Measurements of Melanin Production

**Figure 1:**
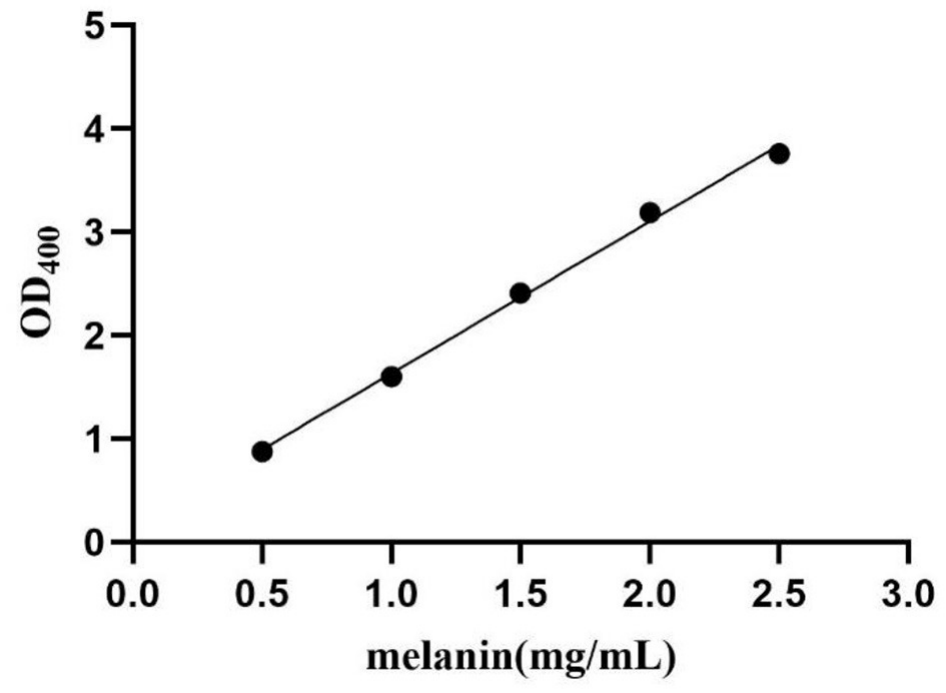
Standard curve of melanin. The formula is Y = 1.471*X + 0.1627, *R*^2^ = 0.9969.

The melanin production of mutant HD-1-Δ*hmgA* was evaluated according to the optical density (OD) under 400 nm. The bacteria were inoculated to 100 mL LB medium and incubated under shaking at 28°C and 200 rpm. The melanin production was quantified by testing the absorbance of the centrifuged culture supernatant at 400 nm (OD400) at different time intervals (Liu et al., 2013; Yang et al., 2018). The melanin yield was calculated using a standard curve based on purified melanin (Sigma Chemical Co.) (Figure 1).

### UV Irradiation of *B. thuringiensis*

2 mL of strain HD-1 and HD-1-ΔhmgA spore-crystal preparations of equal concentration were spread on plates with a diameter of 6 cm and were placed 30 cm below the ultraviolet light (wavelength 254 nm) (Ruan et al., 2004). The irradiation lasts for 0 min, 20 min, 40 min, 60 min, and 80 min respectively. After irradiation, put the preparations into a dark place, then collect and centrifuge at 12000 r/min at 4°C for 10 min, the mixture was used for spores counting and anti-UV survival tests.

Pick single colonies of strain HD-1 and HD-1-Δ*hmgA* onto LB medium (diameter 6 cm) and culture for over 3 days to ensure the crystal proteins were completely released. Place them 30 cm below the ultraviolet light (wavelength 254 nm). The irradiation lasts 0, 60, 120, 180, and 240 min for each sample. Afterward, put them into a dark place, then scratch a proper number of bacteria down, dissolved in ddH_2_O, adjust until they reach the same concentration. These preparations were used for the SDS-PAGE and the insect bioassays. For the SDS-PAGE test, preparations were boiled at 100°C in loading buffer for 10 min, and then loaded onto an 10% acrylamide gel.

### Insect Bioassays

The insect bioassay was carried out on cotton bollworm, *Helicoverpa armigera*. The cotton bollworms were incubated at 30°C for 24 h. The 24-well plates with artificial diet were added 50 μL of protein preparations from strain HD-1 and HD-1-Δ*hmgA* each and repeated three times. Put one cotton bollworm larvae to each well then cultivate at 30°C. After culturing for 3 days, count and calculate the survival rate.

## Results

### Screening of *HmgA*-Deletion Mutant

**Figure 2:**
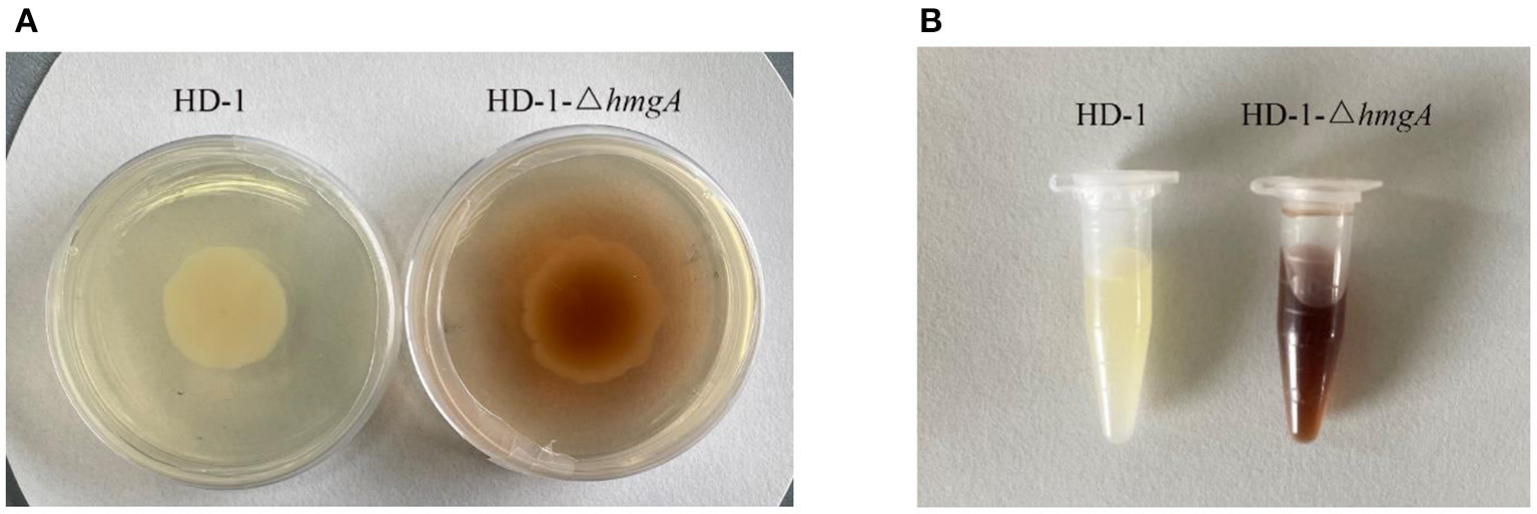
Melanin observation of *B. thuringiensis* HD-1 and HD-1-Δ*hmgA*. **(A)** Strain HD-1 and HD-1-Δ*hmgA* cultured on LB plate for three days. **(B)** Strain HD-1 and HD-1-Δ*hmgA* cultured in liquid LB medium for three days.

**Figure 3:**
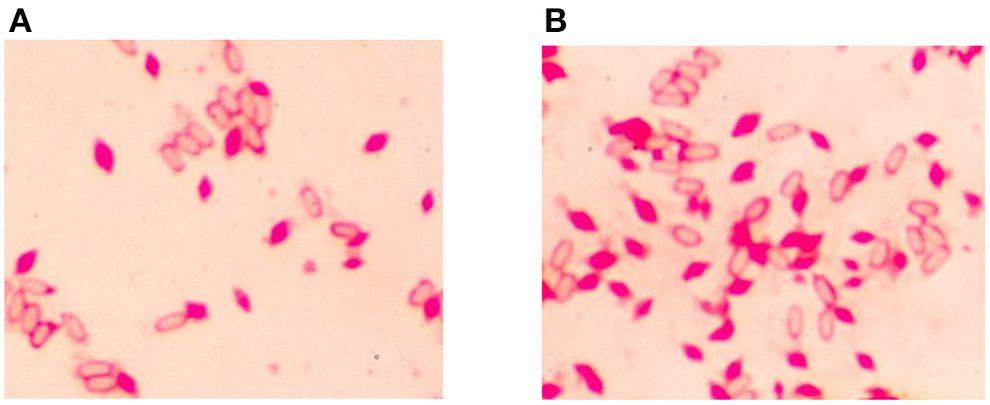
Microscope of *B. thuringiensis* HD-1 and HD-1-Δ*hmgA* under oil lens (100 ×). **(A)** Crystal observation of strain HD-1. **(B)** Crystal observation of mutant HD-1-Δ*hmgA*. Both strains have no distinct difference in spore and crystal formation.

After electroporation, we picked out the transformants onto a new plate and continue to subculture until the brownish colonies appeared. Through PCR amplification and DNA sequencing, the melanin-producing colonies were confirmed *hmgA* knockout mutants. Strain HD-1-Δ*hmgA* began to produce melanin after cultivating for 1 day, and the medium would first become reddish-brown then to dark brown after 2–3 days (Figure 2). The crystal observation of strain HD-1 and HD-1-Δ*hmgA* were done under a microscope. Notably, both strains produce typical diamond-shaped ICPs and have no distinct difference. It means that the deletion of *hmgA* won't affect the formation of ICPs (Figure 3).

### Melanin Yield of HD-1-*ΔHmgA*

**Figure 4:**
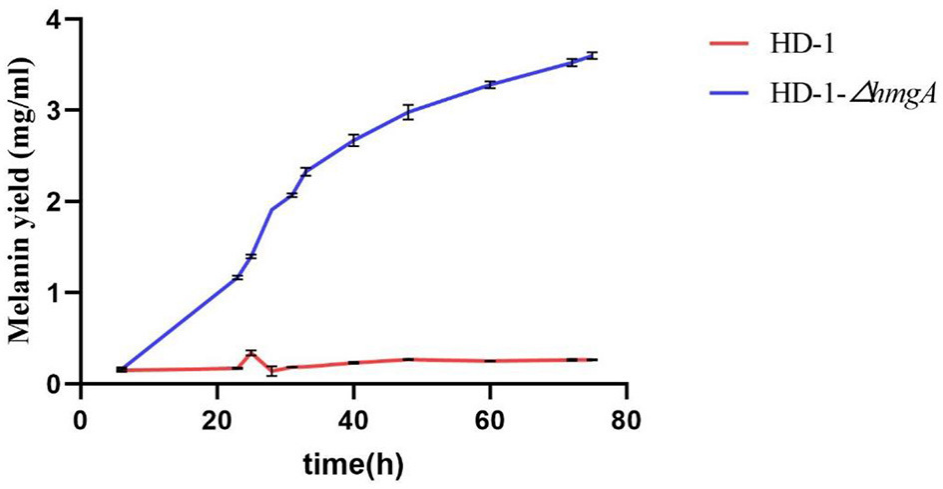
Melanin production curves of *B. thuringiensis* HD-1 and HD-1-Δ*hmgA* in LB medium.

Strains HD-1 and HD-1-Δ*hmgA* were grown in 100 mL LB media at 28°C. The melanin concentrations were determined by the optical density of the culture supernatant at 400 nm after centrifugation. The melanin yield was calculated based on a standard curve performed on purified melanin. Figure 4 showed that strain HD-1-Δ*hmgA* began to produce melanin during the logarithmic phase, and the highest yield reached 3.60 mg/mL after culturing for 3 days. On the contrary, we were unable to detect the formation of melanin in strain HD-1 broth.

### Ultraviolet Test Against HD-1 and HD-1-*ΔHmgA*

**Figure 5:**
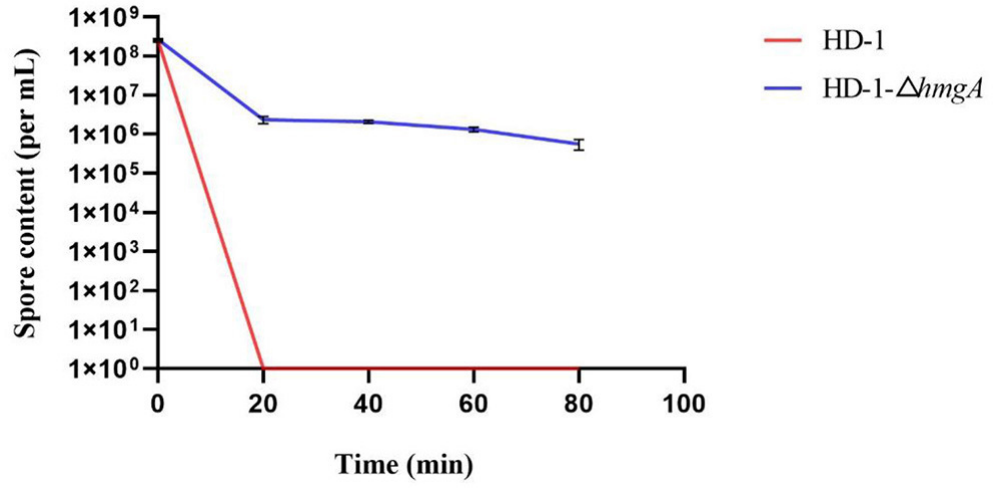
Total spore content after UV irradiation.

Spore-crystal preparations of strain HD-1 and HD-1-Δ*hmgA* were irradiated by UV light under different time intervals. By plate counting, the total spore content of each sample was obtained (Figure 5). As is shown in the figure, before UV irradiation, the total spore contents of both strains were nearly 2 × 10^8^ per mL, having no significant difference. After UV treatment for about 20 min, almost all of the wild-type HD-1 died, while the mutant still had 2 × 10^6^ spores alive. As the irradiation time increased, the spores of the mutant slowly decreased, and there still were 5 × 10^5^ spores surviving after 80 min. Taken together, these results indicated that melanin had a certain protective effect on the bacteria, thereby improving the resistance of spores to UV light.

**Figure 6:**
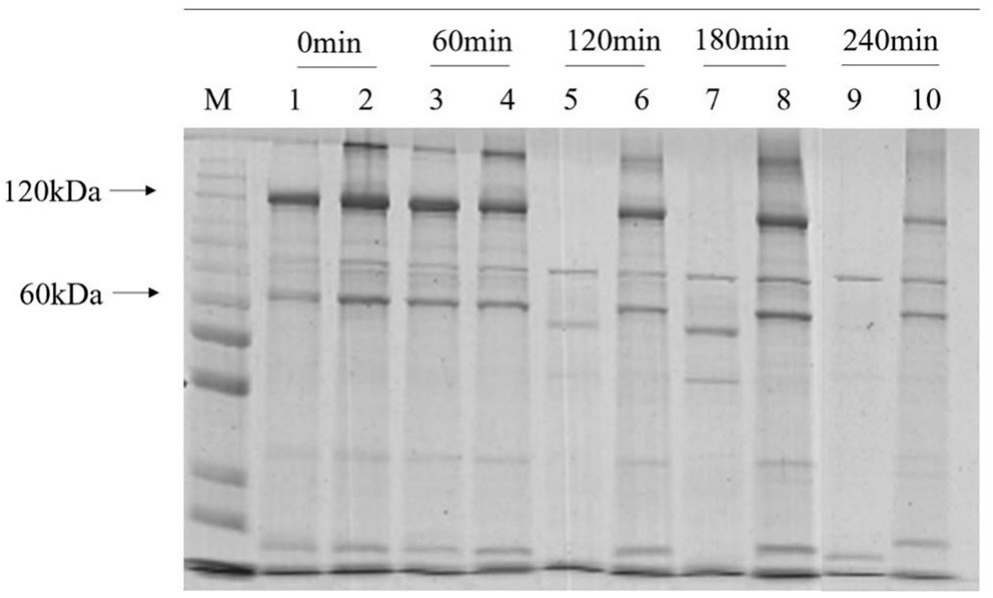
SDS-PAGE of insecticidal proteins of strain HD-1 and HD-1-Δ*hmgA* after UV irradiation. The marker is 200, 150, 120, 100, 85, 70, 60, 50, 40, 30, and 25 kD from top to bottom. Lanes 1, 3, 5, 7, and 9 are wild-type HD-1 under UV treatment for 0, 60, 120, 180, 240 min; lanes 2, 4, 6, 8, and 10 are mutant HD-1-Δ*hmgA* under UV treatment for 0, 60, 120, 180, and 240 min. The arrow marks from top to bottom are several typical Cry proteins.

To detect whether melanin can protect the insecticidal crystal proteins, we performed SDS-PAGE tests on strain HD-1 and HD-1-Δ*hmgA* (Figure 6). The typical ICPs from strain HD-1 are sized 120 kD and 60 kD. The expression level between strain HD-1 and HD-1-Δ*hmgA* differed as the radiation time increased. In the initial states, the protein expressions of both strains were roughly the same (lanes 1, 2), after irradiating for 60 min, the difference was not obvious (lanes 3, 4). But after 120 and 180 min, ICPs from the wild type HD-1 gradually degraded while that of the mutant HD-1-Δ*hmgA* were still quite distinct (lanes 5, 6, and 7, 8). After 240 min of UV treatment, the difference between both strains became more pronounced. All 60 kD proteins of strain HD-1 degraded and that of mutant HD-1-Δ*hmgA* also decreased a bit (lanes 9, 10). The extra bands were considered evidence of the degraded protein. In general, the ICPs expression of mutant HD-1-Δ*hmgA* is less affected by UV irradiation, suggesting that melanin could serve as a protective barrier for ICPs under UV light.

### Insecticidal Properties After UV Irradiation

**Figure 7:**
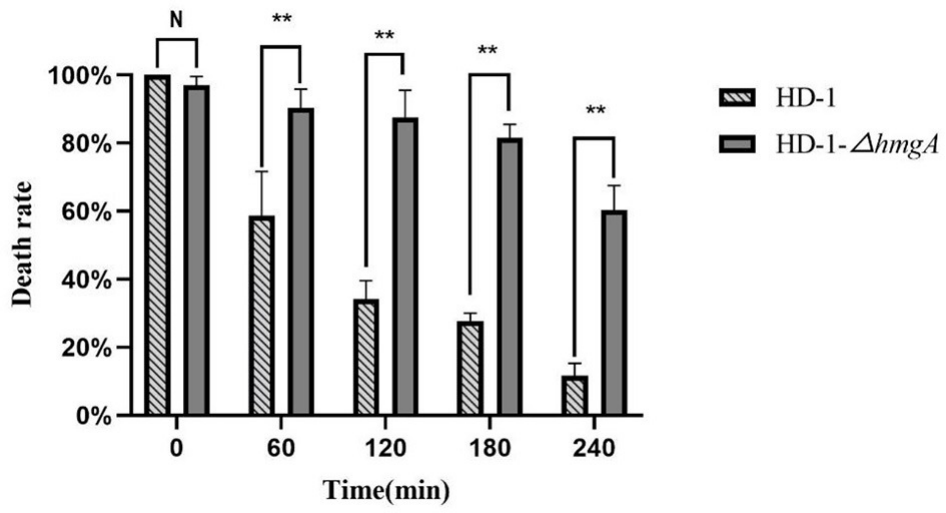
Insecticidal lethality statistics of two strains before and after UV irradiation.

The insecticidal activities of strain HD-1 and HD-1-Δ*hmgA* were assayed on *Helicoverpa armigera* first-instar larvae. The insecticidal lethality statistics are shown in Figure 7. The data showed that the insecticidal properties of both strains were similar before irradiation, reaching nearly 100%. But after 60 min of UV irradiation, the difference between both strains widened. The insect lethality of strain HD-1 was only 60%, which could only kill half of the cotton bollworm, while the mutant still had a 90% insecticidal ability. After UV treatment for 120 and 180 min, the toxicity of strain HD-1-Δ*hmgA* was still quite strong, reaching an over 80% lethality. The toxicity of strain HD-1, however, had been greatly affected and was only poisonous to 20% of the insects. After 240 min of UV irradiation, the insecticidal property of the mutant HD-1-Δ*hmgA* declined a bit, killing nearly 60% of the insects. The above results indicate that the ICPs protected by melanin degraded at a relatively slow rate under UV light so that the mutant HD-1-Δ*hmgA* showed stronger insecticidal activity compared to the wild-type HD-1. It can be inferred that the melanin-producing strain is an ideal light-stable biopesticide and will improve the duration period.

## Discussion

Melanin, a brownish pigment that can be produced naturally by most prokaryotes and eukaryotes, is thought to be a perfect photoprotective reagent against ultraviolet light. Given the fact that the *B. thuringiensis* pesticides are highly vulnerable to UV radiation, many researchers had shed light on the construction of UV-resistance strains using melanin. It is found that *B. thuringiensis* strains can produce melanin in the presence of L-tyrosine at high temperatures (42°C), and a mutant strain BMB181 with high melanin yield was obtained (Ruan et al., 2004; Liu et al., 2013). Later studies suggested that the melanin production in *B. thuringiensis* was related to *hmgA*, and the *hmgA* deletion mutant gained the ability to produce melanin (Yang et al., 2018; Tan et al., 2019). Nevertheless, these strains are crystalliferous with no insecticidal property, thus having limited application potentials.

Given the fact that there are few studies on melanin production in highly toxic strains, in this study, we chose *B. thuringiensis* HD-1 as the target. *B. thuringiensis* HD-1 is an industrial strain with strong toxicity which is highly commercialized and widely used in biopesticides. We used CRISPR/Cas9 to knock out the *hmgA* gene and obtained a melanin-producing mutant in strain HD-1 for the first time. The melanin yield by mutant HD-1-Δ*hmgA* reached 3.60 mg/mL, which is less than the melanin produced by BMB181 (Liu et al., 2013; Yang et al., 2018). This is might because of the various ICPs the mutant HD-1-Δ*hmgA* contains. The results also showed that the deletion of *hmgA* won't affect the cell growth as well as sporulation, and the accumulation of melanin happens in the logarithmic phase of vegetative cells so it wouldn't be a burden on vegetative cells. The ultraviolet test indicates that *B. thuringiensis* spores survived in the presence of melanin, which can also protect the insecticidal proteins from degrading. Mutant HD-1-Δ*hmgA* survived after 80 min of irradiation and had an obvious lower degradation rate of the ICPs. The control line, however, all died after 20 min of UV irradiation and most ICPs degraded after 2 h of UV irradiation. The insect assays suggest that the insecticidal ability of strain HD-1-Δ*hmgA* remained nearly the same after 2 h of irradiation and decreased to 60% in 4 h, while the control line showed little insecticidal property under the same condition. Based on the fact that the mutant HD-1-Δ*hmgA* still had strong insecticidal ability after 3 h of UV irradiation, it can be inferred that when used under field conditions, the improved *B. thuringiensis* preparations will function as expected after 2-day's exposure to sunlight.

Given the wide application of strain HD-1 in pesticides, the mutant we construct can be directly put into production and has great potential to replace the existing industrial strains. This work is a continuation of the previous research from our lab and will contribute to extending the persistence of light-stable biopesticides. At the same time, it also provides a positive reference for the construction and industrialization of UV-tolerant strains with high toxicity. The follow-up research will be carried out in another highly toxic strain YBT-1520. The large-scale fermentation and field trials also need to be carried out. We hope that there will be greater breakthroughs in the development procedure of biopesticides.

## Data Availability Statement

The original contributions presented in the study are included in the article/supplementary material, further inquiries can be directed to the corresponding author/s.

## Author Contributions

LZ, KW, BZ, and MS designed the research. LZ and YC performed the research. LZ, BZ, ZL, and XY analyzed the data. LZ and MS wrote the manuscript. All authors contributed to the article and approved the submitted version.

## Funding

This research was supported by grants from the National Key R&D Program of China (2017YFC204521201) and National Natural Science Foundation of China (31970076).

## Conflict of Interest

The authors declare the following financial interests/personal relationships which may be considered as potential competing interests: Huazhong Agricultural University has applied patents based on this work. The authors of the invention are Ming Sun, Lingyi Zhu, Donghai Peng and Jinshui Zheng.

## Publisher's Note

All claims expressed in this article are solely those of the authors and do not necessarily represent those of their affiliated organizations, or those of the publisher, the editors and the reviewers. Any product that may be evaluated in this article, or claim that may be made by its manufacturer, is not guaranteed or endorsed by the publisher.

## References

[B1] Ahmad S. Lee S. Y. Khan R. Kong H. G. Son G. J. Roy N.. (2017). Identification of a gene involved in the negative regulation of pyomelanin production in *Ralstonia solanacearum*. J. Microbiol. Biotechnol. 27, 1692–1700. 10.4014/jmb.1705.05049 28746990

[B2] Altenbuchner J. (2016). Editing of the *Bacillus subtilis* genome by the CRISPR-Cas9 System. Appl. Environ. Microbiol. 82, 5421–5427. 10.1128/AEM.01453-16 27342565 PMC4988203

[B3] Brar S. K. Verma M. Tyaci R. D. Valero J. R. (2006). Recent advances in downstream processing and formulations of *Bacillus thuringiensis* based biopesticides. Process Biochem. 41, 323–342. 10.1016/j.procbio.2005.07.015

[B4] Cao Z. L. Tan T. T. Jiang K. Mei S. Q. Hou X. Y. Cai J. (2018). Complete genome sequence of *Bacillus thuringiensis* L-7601, a wild strain with high production of melanin. J. Biotechnol. 275, 40–43. 10.1016/j.jbiotec.2018.03.020 29614251

[B5] Chen Y. Deng Y. Wang J. Cai J. Ren G. (2004). Characterization of melanin produced by a wild-type strain of *Bacillus thuringiensis*. J. Gen. Appl. Microbiol. 50, 183–188. 10.2323/jgam.50.183 15754243

[B6] Choi K. Y. (2021). Bioprocess of microbial melanin production and isolation. Front. Bioeng. Biotechnol. 9, 765110. 10.3389/fbioe.2021.765110 34869277 PMC8637283

[B7] Cohen E. Rozen H. Joseph T. Braun S. Margulies L. (1991). Photoprotection of *Bacillus thuringiensis kurstaki* from ultraviolet irradiation. J. Invertebr. Pathol. 57, 343–351. 10.1016/0022-2011(91)90138-G 2066575

[B8] Crickmore N. Zeigler D. R. Feitelson J. Schnepf E. Van Rie J. Lereclus D.. (1998). Revision of the nomenclature for the *Bacillus thuringiensis* pesticidal crystal proteins. Microbiol. Mol. Biol. Rev. 62, 807–813. 10.1128/MMBR.62.3.807-813.1998 9729610 PMC98935

[B9] Jalali E. Maghsoudi S. Noroozian E. (2020). Ultraviolet protection of *Bacillus thuringiensis* through microencapsulation with pickering emulsion method. Sci. Rep. 10, 20633. 10.1038/s41598-020-77721-8 33244110 PMC7691366

[B10] Jallouli W. Sellami S. Sellami M. Tounsi S. (2014). Efficacy of olive mill wastewater for protecting *Bacillus thuringiensis* formulation from UV radiations. Acta Trop. 140, 19–25. 10.1016/j.actatropica.2014.07.016 25093915

[B11] Khasdan V. Ben-Dov E. Manasherob R. Boussiba S. Zaritsky A. (2003). Mosquito larvicidal activity of transgenic *Anabaena* PCC 7120 expressing toxin genes from *Bacillus thuringiensis* subsp. israelensis. FEMS Microbiol. Lett. 227, 189–195. 10.1016/S0378-1097(03)00679-7 14592708

[B12] Kumar S. Chandra A. Pandey K. C. (2008). *Bacillus thuringiensis* (Bt) transgenic crop: an environment friendly insect-pest management strategy. J. Environ. Biol. 29, 641–653. 19295059

[B13] Liu F. Yang W. Ruan L. Sun M. (2013). A *Bacillus thuringiensis* host strain with high melanin production for preparation of light-stable biopesticides. Ann. Microbiol. 63, 1131–1135. 10.1007/s13213-012-0570-0

[B14] Liu Y. T. Sui M. J. Ji D. D. Wu I. H. Chou C. C. Chen C. C. (1993). Protection from ultraviolet irradiation by melanin of mosquitocidal activity of *Bacillus thuringiensis* var. israelensis. J. Invertebr. Pathol. 62, 131–136. 10.1006/jipa.1993.1088 8228318

[B15] Peng R. Xiong A. Li X. Fuan H. Yao Q. (2003). A delta-endotoxin encoded in *Pseudomonas fluorescens* displays a high degree of insecticidal activity. Appl. Microbiol. Biotechnol. 63, 300–306. 10.1007/s00253-003-1343-2 14556036

[B16] Plonka P. M. Grabacka M. (2006). Melanin synthesis in microorganisms–biotechnological and medical aspects. Acta Biochim. Pol. 53, 429–443. 10.18388/abp.2006_3314 16951740

[B17] Rodríguez-Rojas A. Mena A. Martín S. Borrell N. Oliver A. Blázquez J. (2009). Inactivation of the *hmgA* gene of *Pseudomonas aeruginosa* leads to pyomelanin hyperproduction, stress resistance and increased persistence in chronic lung infection. Microbiology 155, 1050–1057. 10.1099/mic.0.024745-0 19332807

[B18] Ruan L. Yu Z. Fang B. He W. Wang Y. Shen P. (2004). Melanin pigment formation and increased UV resistance in *Bacillus thuringiensis* following high temperature induction. Syst. Appl. Microbiol. 27, 286–289. 10.1078/0723-2020-00265 15214633

[B19] Sanchis V. Gohar M. Chaufaux J. Arantes O. Meier A. Agaisse H.. (1999). Development and field performance of a broad-spectrum nonviable asporogenic recombinant strain of *Bacillus thuringiensis* with greater potency and UV resistance. Appl. Environ. Microbiol. 65, 4032–4039. 10.1128/AEM.65.9.4032-4039.1999 10473413 PMC99738

[B20] Sansinenea E. Ortiz A. (2015). Melanin: a photoprotection for *Bacillus thuringiensis* based biopesticides. Biotechnol. Lett. 37, 483–490. 10.1007/s10529-014-1726-8 25381045

[B21] Schnepf E. Crickmore N. Van Rie J. Lereclus D. Baum J. Feitelson J.. (1998). *Bacillus thuringiensis* and its pesticidal crystal proteins. Microbiol. Mol. Biol. Rev. 62, 775–806. 10.1128/MMBR.62.3.775-806.1998 9729609 PMC98934

[B22] Singh S. Nimse S. B. Mathew D. E. Dhimmar A. Sahastrabudhe H. Gajjar A.. (2021). Microbial melanin: recent advances in biosynthesis, extraction, characterization, and applications. Biotechnol. Adv. 53, 107773. 10.1016/j.biotechadv.2021.107773 34022328

[B23] Solano F. (2014). Melanins: skin pigments and much more—types, structural models, biological functions, and formation routes. New J. Sci. 2014, 1–28. 10.1155/2014/498276

[B24] Tan T. T. Zhang X. D. Miao Z. Yu Y. Du S. L. Hou X. Y.. (2019). A single point mutation in *hmgA* leads to melanin accumulation in *Bacillus thuringiensis* BMB181. Enzyme Microb. Technol. 120, 91–97. 10.1016/j.enzmictec.2018.10.007 30396405

[B25] Tran-Ly A. N. Reyes C. Schwarze F. Ribera J. (2020). Microbial production of melanin and its various applications. World J. Microbiol. Biotechnol. 36, 170. 10.1007/s11274-020-02941-z 33043393 PMC7548279

[B26] Turick C. E. Knox A. S. Becnel J. M. Ekechukwu A. A. Milliken C. E. (2010). Properties and function of pyomelanin, in Biopolymers, ed Elnashar M. (Rijeka: InTech), 449–472. 10.5772/10273

[B27] Yang W. Ruan L. Tao J. Peng D. Zheng J. Sun M. (2018). Single amino acid substitution in homogentisate dioxygenase affects melanin production in *Bacillus thuringiensis*. Front. Microbiol. 9, 2242. 10.3389/fmicb.2018.02242 30364256 PMC6193087

[B28] Zhu L. Peng D. Wang Y. Ye W. Zheng J. Zhao C.. (2015). Genomic and transcriptomic insights into the efficient entomopathogenicity of *Bacillus thuringiensis*. Sci. Rep. 5, 14129. 10.1038/srep14129 26411888 PMC4585936

